# Symbol Emergence as an Interpersonal Multimodal Categorization

**DOI:** 10.3389/frobt.2019.00134

**Published:** 2019-12-10

**Authors:** Yoshinobu Hagiwara, Hiroyoshi Kobayashi, Akira Taniguchi, Tadahiro Taniguchi

**Affiliations:** Emergent Systems Laboratory, College of Information Science and Engineering, Ritsumeikan University, Shiga, Japan

**Keywords:** symbol emergence in robotics, multimodal categorization, multiagent system, semiotic communication, language evolution, symbol system, probabilistic generative model

## Abstract

This study focuses on category formation for individual agents and the dynamics of symbol emergence in a multi-agent system through semiotic communication. In this study, the semiotic communication refers to exchanging signs composed of the signifier (i.e., words) and the signified (i.e., categories). We define the generation and interpretation of signs associated with the categories formed through the agent's own sensory experience or by exchanging signs with other agents as basic functions of the semiotic communication. From the viewpoint of language evolution and symbol emergence, organization of a symbol system in a multi-agent system (i.e., agent society) is considered as a bottom-up and dynamic process, where individual agents share the meaning of signs and categorize sensory experience. A constructive computational model can explain the mutual dependency of the two processes and has mathematical support that guarantees a symbol system's emergence and sharing within the multi-agent system. In this paper, we describe a new computational model that represents symbol emergence in a two-agent system based on a probabilistic generative model for multimodal categorization. It models semiotic communication via a probabilistic rejection based on the receiver's own belief. We have found that the dynamics by which cognitively independent agents create a symbol system through their semiotic communication can be regarded as the inference process of a hidden variable in an interpersonal multimodal categorizer, i.e., the complete system can be regarded as a single agent performing multimodal categorization using the sensors of all agents, if we define the rejection probability based on the Metropolis-Hastings algorithm. The validity of the proposed model and algorithm for symbol emergence, i.e., forming and sharing signs and categories, is also verified in an experiment with two agents observing daily objects in the real-world environment. In the experiment, we compared three communication algorithms: no communication, no rejection, and the proposed algorithm. The experimental results demonstrate that our model reproduces the phenomena of symbol emergence, which does not require a teacher who would know a pre-existing symbol system. Instead, the multi-agent system can form and use a symbol system without having pre-existing categories.

## 1. Introduction

Obtaining a computational explanation of a symbol system emerging in the real-world environment is important and it is challenging to understand the mechanism of sharing and forming the symbols that represent the sensory experience as a part of the function of language. Language plays a crucial role in sharing information between people by using semiotic communication. However, how the symbol system of the language has emerged through semiotic communication is still unanswered. In this study, the semiotic communication refers to exchanging signs composed of the signifier (i.e., words) and the signified (i.e., categories). We define the generation and interpretation of signs associated with the categories formed through the agent's own sensory experience or by exchanging signs with other agents as basic functions of the semiotic communication. In order to obtain the computational explanation of a symbol system emerging in the real-world environment, we aim to construct a computational model that comprehensively describes the generation and interpretation of words associated with the categories formed through the agent's own sensory experience or by exchanging words with other agents.

As a study on the origin of words and meanings based on a computational model, Steels (1999) performed the Talking Heads experiment to answer the question: how can a physically embodied autonomous agent arrive at a repertoire of categories for conceptualizing his world and how can a group of such agents ever develop a shared communication system with the same complexity as human natural language. His studies enabled agents to generate categories and develop a shared communication system with syntax in real-world environments. However, simple objects that can be identified by low-dimensional features based on color and shape, such as red circles, blue rectangles, spheres, and cubes, were used in the Talking Heads experiment in order to focus on observing language emergence phenomena.

In contrast, Nakamura et al. (2014) and Taniguchi A. et al. (2016) proposed probabilistic generative models that enable a robot to learn both a language model and a perceptual model for daily objects (e.g., bottles, cups, and cans) and spaces (e.g., toilet, stairs, and elevator) with complex colors and shapes in the living environments. The probabilistic generative models perform multimodal categorization and word discovery using multimodal sensorimotor information obtained by a robot and speech signals from a human instructor. However, these models only explain individual agent learning, and implicitly presume that a human instructor has a fixed and static symbol system.

In this study, we propose a computational model that is constructed by expanding the probabilistic generative model (Nakamura et al., 2014) to a multi-agent system and evaluate the model in an experiment inspired by the Talking Heads experiment (Steels, 1999) using daily objects (e.g., bottles, cups, and cans). The model provides the computational explanation of the symbol emergence in a multi-agent system and the category formation in individual agents through semiotic communication, which is the generation and interpretation of symbols associated with the categories formed using the agent's sensory information. The goal of this paper is to provide a clear view of symbol emergence as an interpersonal multimodal categorization using a computational model that not only categorizes sensory information but also shares the meaning of signs within the multi-agent system.

The main contributions of this paper are as follows:

- We propose a constructive computational model that represents the dynamics of a symbol emergence system by using probabilistic models for multimodal categorization and message passing based on the Metropolis-Hastings (M-H) algorithm. The model represents mutual dependency of individual categorization and formation of a symbol system in a multi-agent system.
- We show that our model representing a multi-agent system and symbol emergence among agents can be regarded as a single agent and a single multimodal categorizer, i.e., an interpersonal categorizer, mathematically. We prove that the symbol emergence in the model is guaranteed to converge.
- We evaluate the proposed model of the symbol emergence and category formation from an experiment by using two agents that can obtain visual information and exchange signs in the real-world environment. The results show the validity of our proposed model.

The rest of this paper is structured as follows. Section 2 describes related works. Section 3 describes the proposed model and inference algorithm for representing the dynamics of symbol emergence and category formation in multi-agent systems. Section 4 presents experimental results, verifying the validity of the proposed model and inference algorithm on the object categorization and symbol emergence. Finally, section 5 presents conclusions.

## 2. Related Works

In studies on language emergence in multi-agent systems, Kirby (1999) showed that the language exchanged between agents involving repeated generation alternation is gradually structured in a simulation model. Morita et al. (2012) showed in simulation experiments that semiotic communication systems emerge from interactions that solve collaborative tasks. Lazaridou et al. (2016) proposed a framework for language learning that relies on multi-agent communication for developing interactive machines (e.g., conversational agents). Lee et al. (2017) proposed a communication game in which two agents, native speakers of their own respective languages, jointly learn to solve a visual referential task. Graesser et al. (2019) proposed a computational framework in which agents equipped with communication capabilities simultaneously play a series of referential games, where agents are trained by deep reinforcement learning. These studies achieved language emergence in multi-agent systems by using a computational model. However, interaction with real-world environments through one's own sensory information without pre-existing categories (i.e., internal representations) was not discussed in these studies.

In studies on language emergence with categorization based on one's own sensory experience, Steels and his group have performed language game experiments using computational models (Steels, 2015). In simulation environments, Steels reported computational experiment in which a group of agents develop ways to identify each other using vocabularies related to spatial concepts (Steels, 1995), and proposed models to examine through which mechanisms autonomous agents could arrive at perceptually grounded categories, such as color (Steels and Belpaeme, 2005). In real-world environments, the Talking Heads experiment (Steels, 1999) is the most widely known setup for grounding a lexicon to a concept based on visual information for the purpose of communication among agents. The experiments demonstrated that the mechanisms worked for lexicon and concept formation on objects (e.g., red circles and blue rectangles) among agents.

The Talking Heads experiment has been improved in various aspects. Robotics sophistication enabled experiments for learning the words and meanings of simple objects and spatial words using mobile robots, such as AIBOs (Steels and Kaplan, 2000; Steels and Loetzsch, 2008). Spranger also performed language game experiments for understanding the evolution of grounded spatial language (Spranger, 2011, 2015), and developed a perceptual system for Sony humanoid robots (Spranger et al., 2012). There also exist studies that improved the Talking Heads experiment regarding the complexity of semantics and grammar (Vogt, 2002, 2005; De Beule et al., 2006; Bleys, 2015; Matuszek, 2018). Vogt (2002) conducted experiments where robots develop a symbolic structure from scratch by engaging in language games, and demonstrated that robots can develop a symbolic structure with which they can communicate the names of a few objects. He also proposed a model for the evolution and induction of compositional structures in the language of a population of simulated robotic agents (Vogt, 2005). These studies based on the Talking Heads experiment focused on symbol grounding with language games and built the foundation of constructive studies on language evolution. However, the experiments were limited to simple objects (e.g., red circles and blue rectangles) and daily objects (e.g., bottles, cups, and cans) with complex colors and shapes found in living environments were not considered.

In contrast, as a study of concept formation and word grounding for daily objects (e.g., bottles, cups, and cans) in the living environment based on the sensory-motor information of a robot, Nakamura et al. (2009) proposed a model for grounding word meanings in multimodal concepts, and Ando et al. (2013) proposed a model of hierarchical object concept formation based on multimodal information. Several methods that enable robots to acquire words and spatial concepts based on sensory-motor information in an unsupervised manner have been proposed (Taniguchi A. et al., 2016; Isobe et al., 2017). Hagiwara et al. (2016, 2018) proposed a Bayesian model to acquire the hierarchical structure of spatial concepts based on the sensory-motor information of a robot in real home environments. Tangiuchi et al. (2019) summarized their studies and related works on cognitive developmental robotics that can learn a language from interaction with their environment and unsupervised learning methods that enable robots to learn a language without hand-crafted training data. As studies on developmental robotics (Cangelosi and Schlesinger, 2014), Cangelosi and his group have proposed computational models for an iCub humanoid robot to ground action words through embodied communications (Marocco et al., 2010; Stramandinoli et al., 2017; Taniguchi et al., 2017; Zhong et al., 2019). Marocco et al. (2010) proposed a computational model that enables the iCub humanoid robot to learn the meaning of action words by physically interacting with the environment and linking the effects of actions with the behavior observed on an object before and after the action. Stramandinoli et al. (2017) proposed a robotic model for grounding abstract action words (i.e., USE, MAKE) through the hierarchical organization of terms directly linked to perceptual and motor skills of the iCub humanoid robot. These studies focused on language acquisition by a robot from a person who gives speech signals to the robot, and enables robots to discover words and categories based on their embodied meanings from raw sensory-motor information (e.g., visual, haptic, auditory, and acoustic speech information) in the living environment. They presume that a person has knowledge about categories and signs representing the categories, i.e., a symbol system shared in the society. Therefore, these computational models cannot be considered as a constructive model of symbol emergence systems. These studies have not dealt with the dynamics of emerging symbols while agents form categories based on sensory-motor information.

**Figure 1:**
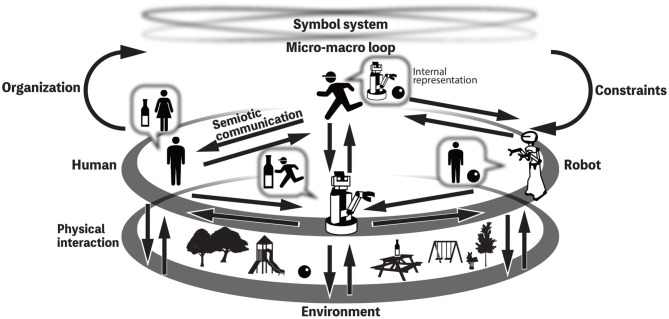
Overview of the symbol emergence systems (Taniguchi T. et al., 2016).

Taniguchi T. et al. (2016) and Taniguchi et al. (2018) introduced a concept of *symbol emergence system*, which is a multi-agent system that dynamically organizes a symbol system, for example, by physical interaction with the environment and semiotic communication with other agents as shown in Figure 1. The figure represents a symbol emergence system. Note that a symbol system cannot be controlled by anyone, but all individuals are constrained by an emergent and shared symbol system. In addition, all of them contribute to creating the socially shared symbol system. To understand this phenomena, the coupled dynamics of both a symbol system shared between the agents and the internal representation systems of individuals has to be modeled with a constructive and computational approach. A computational model for category formation and lexical acquisition by a robot has been proposed and evaluated using daily objects (e.g., bottles, cups, and cans) in the living environment (Nakamura et al., 2014). However, thus far, there is a lack of a computational model that would describe the mutual dependency on symbol emergence and would be evaluated in experiments like the Talking Heads experiment (Steels, 1999).

In this study, we explicitly modeled the dynamics of multi-agent communication and categorization of each agent as a symbol emergence system. By modeling the categorization of each agent and the communication of multi-agents as a symbol emergent system, the mechanism of symbol emergence can be explained as the inference process of model parameters, and an approximate solution is guaranteed by a statistical inference algorithm (e.g., M-H algorithm and Gibbs sampling). Mathematical explanations and guarantees of our model in the categorization of each agent and symbol emergence of multi-agent systems are described in Chapter 3, and how our model works in an experiment likes the talking head experiment is described in Chapter 4. The experiment demonstrates the process in which words representing object categories are shared among agents in a language game using three communication algorithms.

## 3. Proposed Model and Inference Algorithm

### 3.1. Overview

Conventionally, several studies on concept acquisition based on multimodal information using a Bayesian generative model has been performed. Nakamura et al. (2009) proposed a model in which a robot acquires the concept of an object using a multimodal latent Dirichlet allocation (mLDA) consisting of image, sound, haptic, and language information obtained through human linguistic-instructions. The authors also proposed a spatial concept acquisition model consisting of position, image, and language information (Hagiwara et al., 2016, 2018). These studies model concept acquisition based on multimodal information including language information within an individual agent. These models are effective in concept formation and language acquisition based on linguistic instructions by a person who has a stable language system. However, there was a problem how to model the language emergence through communications between agents who don't have stable language systems. As a solution for this problem, this study proposes a model that considers multiple agents as a symbol emergence system. This model makes it possible to explain the process of symbol emergence based on the concept formation and the language communication between agents as an inference process of a word by the M-H algorithm.

**Figure 2:**
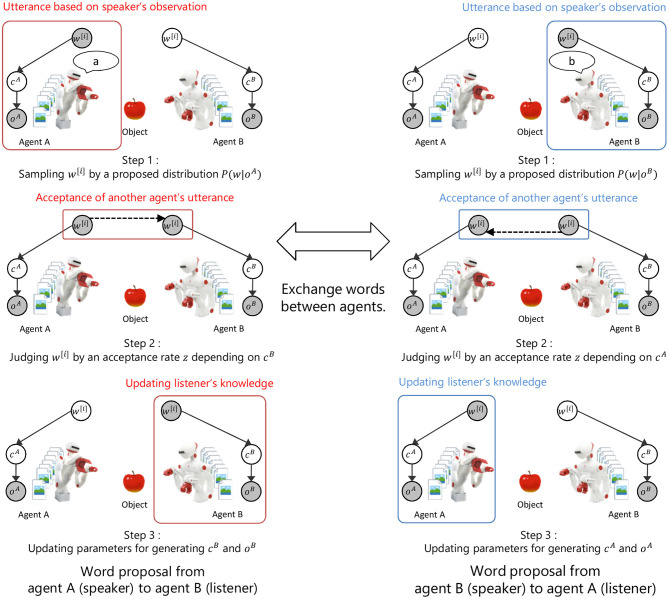
Overview of the proposed model and inference process. *c*^*A*^ and *c*^*B*^ show the index of a category in agent A and B. *o*^*A*^ and *o*^*B*^ show observations in agent A and B. *w* shows the index of a word. Symbol emergence system based on categorization and communication is modeled as inference process of a word between agents by M-H algorithm.

Figure 2 shows the overview of the proposed model and the inference process. In the proposed model, the concept formation in an individual agent and the language communication between agents are modeled as a symbol emergence system. The inference process of a word *w*^[*i*]^ is as follows.

- Step 1: sampling a word *w*^[*i*]^ from the proposal distribution of words based on an observation *o*^*A*^ in agent A.
- Step 2: probably accepting the proposed word *w*^[*i*]^ based on the acceptance rate *z* calculated from the model parameters of agent B.
- Step 3: updating the model parameters of agent B when the proposed word *w*^[*i*]^ is accepted.
- Steps 1 to 3 are repeated by changing role between agents.

In this model, a word *w*^[*i*]^ is a latent variable in the symbol emergence system, but can be interpreted as an observation given from another agent in the inference process. In step 1, the sampling of a word from the proposal distribution based on model parameters of the speaker agent can be interpreted as an utterance based on speaker's observation. In step 2, the probabilistic acceptance of a proposed word based on the model parameters of the listener agent can be interpreted as the acceptance of another agent's utterance based on the listener's knowledge. In step 3, the update of model parameters in the listener agent based on accepted words can be interpreted as the update of the listener's knowledge by accepting the utterance of another person. Thus, the proposed model and the inference algorithm explain the exchange of words between agents based on observations as the model inference process. The details of the proposed model and the inference algorithm are explained in the following sections.

### 3.2. Expansion of a Multimodal Categorizer From Personal to Interpersonal

The computational model that we propose in this paper is based on a key finding that a probabilistic generative model of multimodal categorization can be divided into several sub-modules of probabilistic generative models for categorization and message passing between the sub-modules. This idea of dividing a large probabilistic generative model for developing cognitive agents and re-integrating them was firstly introduced as a SERKET framework (Nakamura et al., 2018). However, their idea was only applied to creating a single agent. We found that the idea can be used for modeling multi-agent systems and is very suitable for modeling dynamics of a symbol emergence system.

**Figure 3:**
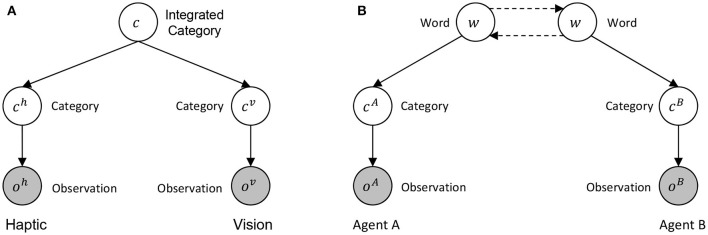
The expansion of a multimodal categorizer from personal to interpersonal: **(A)** shows a generative model of a personal multimodal categorizer between haptics and vision, and **(B)** shows a generative model of an inter-personal multimodal categorizer between the agents. Dashed lines in **(B)** show communication between agents. The parameters of these models are simplified.

We modeled the symbol emergence in a multi-agent system and the category formation in individual agents as a generative model by expanding a personal multimodal categorizer (see Figure 3A) to an interpersonal multimodal categorizer (see Figure 3B). First, (A) shows a personal multimodal categorizer, which is a generative model with an integrated category *c* as a latent variable and sensor information from haptics and vision as observations *o*^*h*^ and *o*^*v*^. The model is a simple version of multimodal latent Dirichlet allocation used as an object categorizer in the previous studies (Nakamura et al., 2009; Ando et al., 2013). Next, (B) shows an interpersonal multimodal categorizer in which two agents are modeled as a collective intelligence, with word *w* as a latent variable, and sensor information from agent A and B as observations *o*^*A*^ and *o*^*B*^. As shown in Figure 3, the model generating observations through categories on each sensor from an integrated concept in an agent can be extended as the model generating observations through categories on each agent from a word in a multi-agent system.

Figure 3A represents a graphical model for probabilistic generative model multimodal categorization (e.g., Nakamura et al., 2014). It can integrate multimodal information, e.g., haptics and visual information, and form categories. Index of category is represented by *c* in this figure. Following the SERKET framework (Nakamura et al., 2018), we can divide the model into two modules and a communication protocol for a shared node. Here, *c* is shared by the two modules and the node is renamed by *w*. We regard an index *w* as an index of word. In this case, if we regard the two separated modules as two individual agents (i.e., agent A and agent B), the communication between the two nodes can be considered as exchange of signs (i.e., words). As we see later, we found that, if we employ the Metropolis-Hastings algorithm, which is one of the communication protocols that the original SERKET paper proposed, the communication protocol between the nodes can be considered as semiotic communication between two agents. Roughly speaking, the communication is described as follows. Agent A recognizes an object and generates words for Agent B. If the word is consistent to the belief of Agent B, then Agent B accepts the naming with a certain probability; otherwise, Agent B rejects the information, i.e., does not believe the meaning. If the rejection and acceptance probability of the communication is the same as the probability of the M-H algorithm, the posterior distribution over *w*, i.e., symbol emergence among the agents, is theoretically the same as the posterior distribution over *c*, i.e., interpersonal categorization.

### 3.3. Generative Process on the Interpersonal Multimodal Categorizer

**Figure 4:**
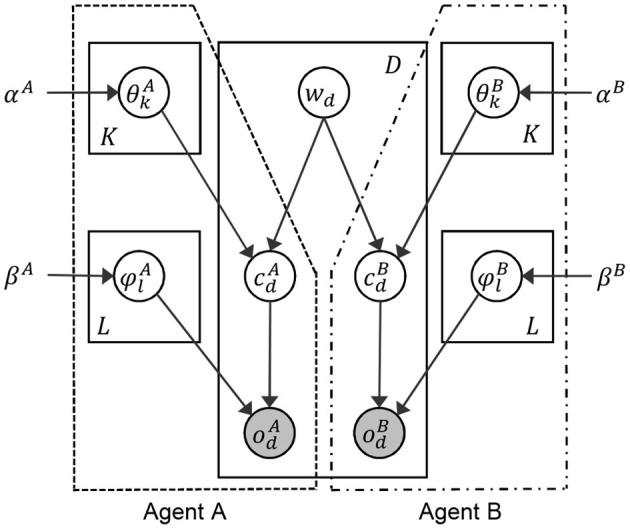
Graphical model of the proposed interpersonal multimodal categorizer.

This subsection describes the generative process of the interpersonal multimodal categorizer. Figure 4 shows the graphical model is a single graphical model. However, following the SERKET framework (see Figure 3), it can be owned by two different agents separately. The right and left parts indicated with a dashed line in Figure 4 show the parts owned by agents A and B, respectively. Figure 4 and Table 1 show the graphical model and the parameters of a proposed interpersonal multimodal categorizer, respectively.

**Table 1:** Definition of variables in the proposed interpersonal multimodal categorizer.

*w*_*d*_	Index of the word
$c_{d}^{A},c_{d}^{B}$	Index of the category
$o_{d}^{A},o_{d}^{B}$	Observations of agents A and B
$\phi_{l}^{A},\phi_{l}^{B}$	Parameters of the multinomial distribution
$\theta_{k}^{A},\theta_{k}^{B}$	Parameters of the multinomial distribution
α, β	Hyperparameters for θ, ϕ
*K*	Number of words
*L*	Number of categories
*D*	Number of data points

Index *w*_*d*_ (of word *w*) connects the agents A and B as a hidden variable to generate the index of a category *c*_*d*_ from the parameter of multinomial distribution θ_*k*_ in each agent. $o_{d}^{A}$ and $o_{d}^{B}$ are observations on data point *d* obtained from the sensors attached to the agents A and B, respectively. $c_{d}^{A}$ and $c_{d}^{B}$ are indices of a category allocated to an observation $o_{d}^{A}$ and $o_{d}^{B}$, respectively. $\phi_{l}^{A}$ and $\phi_{l}^{B}$ are the parameters of multinomial distributions to generate observations $o_{d}^{A}$ and $o_{d}^{B}$ based on categories $c_{d}^{A}$ and $c_{d}^{B}$. α and β are the hyperparameters of θ and ϕ. *K* is the number of words in the word dictionary that a robot has. *L* is the number of categories. *D* is the number of observed data points. The multinomial distribution is denoted as Multi(·), and the Dirichlet distribution is denoted as Dir(·).

The generative process of the interpersonal multimodal categorizer is described as follows.

The parameters $\phi_{l}^{A}$ and $\phi_{l}^{B}$ of multinomial distributions on each category (*l* ∈ *L*) are shown as follows:

(1) $$\begin{array}{l} \phi_{l}^{A}\sim \text{Dir}\left(\beta^{A}\right), \end{array}$$

(2) $$\begin{array}{l} \phi_{l}^{B}\sim \text{Dir}\left(\beta^{B}\right). \end{array}$$

The parameters $\theta_{k}^{A}$ and $\theta_{k}^{B}$ of multinomial distributions on each word (*k* ∈ *K*) are shown as follows:

(3) $$\begin{array}{l} \theta_{k}^{A}\sim \text{Dir}\left(\alpha^{A}\right), \end{array}$$

(4) $$\begin{array}{l} \theta_{k}^{B}\sim \text{Dir}\left(\alpha^{B}\right). \end{array}$$

The following operations from (5) to (8) are repeated for each data point (*d* ∈ 1, 2, …, *D*):

- Observations $o_{d}^{A}$ and $o_{d}^{B}$ generated from categories $c_{d}^{A}$ and $c_{d}^{B}$ are shown as follows:

(5) $$\begin{array}{l} o_{d}^{A}\sim \text{Multi}\left(\phi_{c_{d}^{A}}^{A}\right), \end{array}$$

(6) $$\begin{array}{l} o_{d}^{B}\sim \text{Multi}\left(\phi_{c_{d}^{B}}^{B}\right). \end{array}$$

- Indices of categories $c_{d}^{A}$ and $c_{d}^{B}$ generated from word *w*_*d*_ are shown as follows:

(7) $$\begin{array}{l} c_{d}^{A}\sim \text{Multi}\left(\theta_{w_{d}}^{A}\right), \end{array}$$

(8) $$\begin{array}{l} c_{d}^{B}\sim \text{Multi}\left(\theta_{w_{d}}^{B}\right). \end{array}$$

Theoretically, the generative model is a type of pre-existing model for multimodal categorization (Nakamura et al., 2014, 2018) for an individual agent. In this study, we designed the generation process of the interpersonal multimodal categorizer by interpreting the observations in the multiple sensors of an agent in pre-existing model as the observations in sensors of multi-agents. Expansions in the inference process for the interpersonal multimodal categorizer are described in the next subsection.

### 3.4. Communication Protocol as an Inference Algorithm on the Interpersonal Multimodal Categorizer

This subsection describes the protocol of semiotic computation between two agents and cognitive dynamics of categorization in individual agents. As a whole, the total process can be regarded as a model of symbol emergence in the multi-agent system. Additionally, the total process can be regarded as an inference process of the probabilistic generative model integrating the two agents' cognitive systems (Figure 4).

#### 3.4.1. Gibbs Sampling

First, to introduce our proposed model, we describe an ordinary Gibbs sampling algorithm for the integrative probabilistic generative model in Figure 4. Gibbs sampling algorithm is widely used for multimodal categorization and language learning in robotics. Gibbs sampling (Liu, 1994) is known as a type of Markov chain Monte Carlo (MCMC) algorithm for inferring latent variables in probabilistic generative models.

Algorithm 1 shows the inference algorithm on the model of Figure 4 using Gibbs sampling. In the algorithm 1, *i* shows the number of iterations; **O**^*A*^ and **O**^*B*^ denote a set of all observations in agents A and B, respectively; **C**^*A*^ and **C**^*B*^ denote a set of all categories in agents A and B, respectively; and *W* denotes a set of all words. In line 14 of Algorithm 1, word $w_{d}^{\left[i\right]}$ is sampled from the product of probability distributions $P\left(c_{d}^{A\left[i\right]}|\theta_{k}^{A\left[i\right]}\right)$ and $P\left(c_{d}^{B\left[i\right]}|\theta_{k}^{B\left[i\right]}\right)$ based on parameters $\theta_{k}^{A\left[i\right]}$ and $\theta_{k}^{B\left[i\right]}$ in agents A and B.

If an agent can observe both $\theta_{k}^{A\left[i\right]}$ and $\theta_{k}^{B\left[i\right]}$, which are internal representations of each agent, this algorithm can work. However, Agent A cannot observe $\theta_{k}^{B\left[i\right]}$, or Agent B cannot observe $\theta_{k}^{A\left[i\right]}$. Therefore, no agent can perform Gibbs sampling in this multi-agent system. In this sense, this is not a valid cognitive model for representing the symbol emergence between two agents.

**Algorithm 1:** Gibbs sampling algorithm

1: Initialize all parameters
2: **for** *i* = 1 to *I* **do**
3: **for** *l* = 1 to *L* **do**
4: $\phi_{l}^{A\left[i\right]}\sim \text{Dir}\left(\phi_{l}^{A\left[i\right]}|{\text{O}}^{A},{\text{C}}^{A\left[i-1\right]},\beta^{A}\right)$
5: $\phi_{l}^{B\left[i\right]}\sim \text{Dir}\left(\phi_{l}^{B\left[i\right]}|{\text{O}}^{B},{\text{C}}^{B\left[i-1\right]},\beta^{B}\right)$
6: **end for**
7: **for** *k* = 1 to *K* **do**
8: $\theta_{k}^{A\left[i\right]}\sim \text{Dir}\left(\theta_{k}^{A\left[i\right]}|{\text{O}}^{A},{\text{W}}^{\left[i-1\right]},\alpha^{A}\right)$
9: $\theta_{k}^{B\left[i\right]}\sim \text{Dir}\left(\theta_{k}^{B\left[i\right]}|{\text{O}}^{B},{\text{W}}^{\left[i-1\right]},\alpha^{B}\right)$
10: **end for**
11: **for** *d* = 1 to *D* **do**
12: $c_{d}^{A\left[i\right]}\sim \text{Multi}\left(c_{d}^{A\left[i\right]}|\theta_{w_{d}^{\left[i-1\right]}}^{A\left[i\right]}\right)\text{Multi}\left(o_{d}^{A}|\phi_{c_{d}^{A\left[i\right]}}^{A\left[i\right]}\right)$
13: $c_{d}^{B\left[i\right]}\sim \text{Multi}\left(c_{d}^{B\left[i\right]}|\theta_{w_{d}^{\left[i-1\right]}}^{B\left[i\right]}\right)\text{Multi}\left(o_{d}^{B}|\phi_{c_{d}^{B\left[i\right]}}^{B\left[i\right]}\right)$
14: $w_{d}^{\left[i\right]}\sim \text{Multi}\left(c_{d}^{A\left[i\right]}|\theta_{w_{d}^{\left[i\right]}}^{A\left[i\right]}\right)\text{Multi}\left(c_{d}^{B\left[i\right]}|\theta_{w_{d}^{\left[i\right]}}^{B\left[i\right]}\right)$
15: **end for**
16: **end for**

#### 3.4.2. Computational Model of the Symbol Emergence Based on an Inference Procedure Using the Metropolis-Hastings Algorithm

A communication protocol based on the M-H algorithm proposed in SERKET enables us to develop a valid cognitive model, i.e., updating parameters by an agent does not require the agent to use cognitively unobservable information (Hastings, 1970; Nakamura et al., 2018). The M-H algorithm is one of Markov chain Monte Carlo algorithms, and Gibbs sampling is a special case of it. It is known that both algorithms can sample latent variables from the posterior distribution. That means that, theoretically, both of the algorithms can converge to the same stationary distribution.

Algorithm 2 shows the proposed inference algorithm based on the M-H algorithm. It can be also regarded as a semiotic communication between two agents, and individual object categorization process under the influence of words that are given by the other agent.

**Algorithm 2:** Proposed interactive learning process based on the M-H algorithm

1: Initialize all parameters
2: **for** *i* = 1 to *I* **do**
3: **W**^[*i*]^, **C**^*A*[*i*]^, **C**^*B*[*i*]^, θ^*B*[*i*]^ = M-H algorithm(**O**^*A*^,**W**^*A*[*i*−1]^, **C**^*A*[*i*−1]^,**O**^*B*^,**W**^*B*[*i*−1]^,**C**^*B*[*i*−1]^,θ^*B*[*i*−1]^)
4: **W**^*B*[*i*]^ ← **W**^[*i*]^
5: **W**^[*i*]^, **C**^*B*[*i*]^, **C**^*A*[*i*]^, θ^*A*[*i*]^ = M-H algorithm(**O**^*B*^,**W**^*B*[*i*]^,**C**^*B*[*i*]^, **O**^*A*^,**W**^*A*[*i*−1]^,**C**^*A*[*i*]^,θ^*A*[*i*−1]^)
6: **W**^*A*[*i*]^ ← **W**^[*i*]^
7: **end for**

A set of all the words *W*^[*i*]^ at *i*_*th*_ iteration is calculated by two steps of the M-H algorithm, as shown in lines 3 and 5. Basically, the M-H algorithm requires information that an agent can observe within the dotted line in Figure 4 and *w*_*d*_. In this model of symbol emergence, word *w*_*d*_ is generated, i.e., uttered, by a speaker agent, either A or B. A listener agent judges if the word properly represents the object the agent looks at. The criterion for the judgement should rely on the information the listener knows, i.e., the probability variables inside the dotted line in Figure 4.

Algorithm 3 shows the M-H algorithm, where *Sp* and *Li* are the speaker and listener, respectively. Generation of word *w*_*d*_ from speaker's observation $o_{d}^{Sp}$ and category $c_{d}^{Sp}$, which the speaker regards as the target object, is modeled as a sampling process using $P\left(w_{d}^{Sp}|c_{d}^{Sp},\theta_{d}^{Sp}\right)$. This sampling can be performed by using the information that is available to the speaker agent. At line 3 in Algorithm 2, the sampling and judgment of words *W* are performed with agent A as the speaker, and agent B as the listener. At line 5 in Algorithm 2, the sampling and judgment of words *W* are performed with agent B as the speaker, and agent A as the listener. In the M-H algorithm, the listener agent can update its parameters by using information that is available to the listener agents, i.e., ·^*Li*^ and *w*_*d*_.

**Algorithm 3:** M-H algorithm

1: M-H algorithm(**O**^*Sp*^, **W**^*Sp*^, **C**^*Sp*^, **O**^*Li*^, **W**^*Li*^, **C**^*Li*^, θ^*Li*^):
2: **for** *l* = 1 to *L* **do**
3: $\phi_{l}^{Sp}\sim \text{Dir}\left(\phi_{l}^{Sp}|{\text{O}}^{Sp},{\text{C}}^{Sp},\beta^{Sp}\right)$
4: **end for**
5: **for** *k* = 1 to *K* **do**
6: $\theta_{k}^{Sp}\sim \text{Dir}\left(\theta_{k}^{Sp}|{\text{O}}^{Sp},{\text{W}}^{Sp},\alpha^{Sp}\right)$
7: **end for**
8: **for** *d* = 1 to *D* **do**
9: $c_{d}^{Sp}\sim \text{Multi}\left(c_{d}^{Sp}|\theta_{k}^{Sp}\right)\text{Multi}\left(o_{d}^{Sp}|\phi_{l}^{Sp}\right)$
10: **end for**
11: **for** *d* = 1 to *D* **do**
12: $w_{d}^{Sp}\sim P\left(w_{d}^{Sp}|c_{d}^{Sp},\theta_{k}^{Sp}\right)$
13: $z\sim \text{min}\left(1,\frac{P\left(c_{d}^{Li}|\theta_{k}^{Li},w_{d}^{Sp}\right)}{P\left(c_{d}^{Li}|\theta_{k}^{Li},w_{d}^{Li}\right)}\right)$
14: *u* ~ Unif(0, 1)
15: **if** *u* ≤ *z* **then**
16: $w_{d}=w_{d}^{Sp}$
17: **else**
18: $w_{d}=w_{d}^{Li}$
19: **end if**
20: **end for**
21: **for** *l* = 1 to *L* **do**
22: $\phi_{l}^{Li}\sim \text{Dir}\left(\phi_{l}^{Li}|{\text{O}}^{Li},{\text{C}}^{Li},\beta^{Li}\right)$
23: **end for**
24: **for** *k* = 1 to *K* **do**
25: $\theta_{k}^{Li}\sim \text{Dir}\left(\theta_{k}^{Li}|{\text{O}}^{Li},W,\alpha^{Li}\right)$
26: **end for**
27: **for** *d* = 1 to *D* **do**
28: $c_{d}^{Li}\sim \text{Multi}\left(c_{d}^{Li}|\theta_{k}^{Li}\right)\text{Multi}\left(o_{d}^{Li}|\phi_{l}^{Li}\right)$
29: **end for**
30: **return** **W**, **C**^*Sp*^, **C**^*Li*^, θ^*Li*^

Simultaneous use of Algorithms 2 and 3 performs a probabilistic inference of the probabilistic generative models shown in Figure 4. Importantly, the M-H algorithm can sample words from the posterior distribution exactly the same way as Gibbs sampling that requires all information owned by an individual agent in a distributed manner. This gives us a mathematical support of the dynamics of symbol emergence.

#### 3.4.3. Dynamics of Symbol Emergence and Category Formation

**Figure 5:**
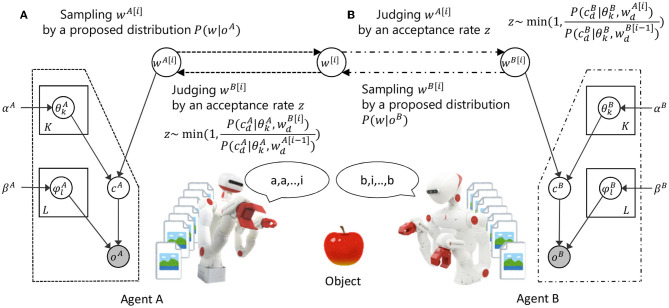
Dynamics of symbol emergence and category formation through semiotic communication between the agents in the proposed method.

This subsection describes how the proposed inference algorithm explains the dynamics of symbol emergence and category formation through semiotic communication. Figure 5 conceptually shows the relationship between the dynamics of symbol emergence and concept formation between the agents, and the inference process for a word in the proposed model. The proposed model consists of the categorization part, where the agents form categories individually, and the communication part, in which the agents exchange words between them. The categorization part is modeled based on latent Dirichlet allocation (LDA). The communication part connects the categorization parts of agents A and B. We modeled the communication part as the inference process of hidden variable $w_{d}^{\left[i\right]}$ in the M-H algorithm.

In line 12 of Algorithm 3, word $w_{d}^{Sp}$ is sampled by a proposal distribution with parameters of the speaker only (i.e., $c_{d}^{Sp}$ and $\theta_{k}^{Sp}$) by the following formula:

(9) $$\begin{array}{l} w_{d}^{Sp}\sim P\left(w_{d}^{Sp}|c_{d}^{Sp},\theta_{k}^{Sp}\right). \end{array}$$

The process can be regarded as a word utterance from agent A in observation $o_{d}^{A}$ based on its internal parameters, as shown in Figure 5A. This is a part of semiotic communication.

In line 13, sampled word $w_{d}^{Sp}$ is judged by the listener by using acceptance rate *z* calculated by the following formula:

(10) $$\begin{array}{l} z\sim \text{min}\left(1,\frac{P\left(c_{d}^{Li}|\theta_{k}^{Li},w_{d}^{Sp}\right)}{P\left(c_{d}^{Li}|\theta_{k}^{Li},w_{d}^{Li}\right)}\right). \end{array}$$

Acceptance rate *z* of sampled word $w_{d}^{Sp}$ can be calculated from parameters of the listener only (i.e., $c_{d}^{Li},\theta_{k}^{Li},w_{d}^{Li}$). Therefore, this is plausible from a cognitive perspective.

In lines 14–19, sampled word $w_{d}^{Sp}$ is probabilistically accepted or rejected by the listener using acceptance rate *z* and uniform random number *u* by the following formulas:

(11) $$\begin{array}{l} u\sim \text{Unif}\left(0,1\right), \end{array}$$

(12) $$w_{d}^{\left[i\right]}=\{\begin{array}{ll} w_{d}^{Sp[i]} & \left(u\leq z\right)\\ w_{d}^{Li[i-1]} & (otherwise), \end{array}$$

where the continuous uniform distribution is denoted as Unif(·). Word $w_{d}^{Lp\left[i-1\right]}$ of the listener at a previous iteration is used when sampled word $w_{d}^{Sp}$ is rejected. Roughly speaking, if the listener agent considers that the current word is likely to be the word that represents the object that the listener also looks at, the listener agent accepts the word and updates its internal representations with a high probability. The process can be explained as a judgment as to whether agent B accepts or rejects an utterance of agent A based on self-knowledge, as shown in Figure 5B.

In lines 21–29, the internal parameters of the listener are updated based on judged words *W* by the following formulas:

(13) $$\begin{array}{l} \phi_{l}^{Li}\sim \text{Dir}\left(\phi_{l}^{Li}|{\text{O}}^{Li},{\text{C}}^{Li},\beta^{Li}\right), \end{array}$$

(14) $$\begin{array}{l} \theta_{k}^{Li}\sim \text{Dir}\left(\theta_{k}^{Li}|{\text{O}}^{Li},\text{W},\alpha^{Li}\right), \end{array}$$

(15) $$\begin{array}{l} c_{d}^{Li}\sim \text{Multi}\left(c_{d}^{Li}|\theta_{w_{d}}^{Li}\right)\text{Multi}\left(o_{d}^{Li}|\phi_{c_{d}^{Li}}^{Li}\right). \end{array}$$

The process can be explained as the updation of self-knowledge based on partial acceptance of the other agent's utterance. Because both the utterance and acceptance of words use only self-knowledge, these processes can be rationally convinced.

As shown in Algorithm 2, words *W*, categories *C* and parameters ϕ_*l*_ and θ_*k*_ are inferred by repeating this process with *I* iterations while exchanging the agents A and B. This inference process not only is rationalized as a model of the symbol emergence and category formation through semiotic communication between the agents, but also gives a mathematical guarantee on the inference of the model parameters. Details of the calculation process on the inference of model parameters by the M-H algorithm are described in the Supplementary Material.

## 4. Experiment

### 4.1. Experimental Setup

#### 4.1.1. Procedure

**Figure 6:**
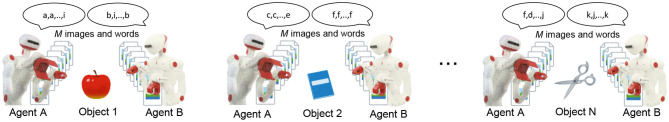
Overview of the experiment: agents A and B observed *N* objects placed at the front of them. Both agents capture images and suggest words *M* times for each object.

We performed an experiment to verify the validity of the proposed model and algorithm for modeling the dynamics of symbol emergence and concept formation. Specifically, we used an experiment of object categorization in the real world. We also discuss the functions required for semiotic communication in the category formation and the symbol emergence in multi-agent systems from the comparison of three communication algorithms on the proposed model. Figure 6 shows an overview of the experiment.

The experiment was performed by the following procedure:

- Step 1: Capture and memorize *N* objects with *M* images for each object on agent A and B with different angles.
- Step 2: Convert a memorized image to a visual feature as observations $o_{d}^{A},o_{d}^{B}$ for agent A and B.
- Step 3: Sampling *w*_*d*_ and updating model parameters from observations $o_{d}^{A},o_{d}^{B}$ by the M-H algorithm. This step corresponds to semiotic communication between agents A and B based on the opponent's utterances and self-organized categories.
- Step 4: Repeat step 3 with *I* iterations.
- Step 5: Evaluate the coincidence of words and categories between agents A and B for each object.

In the experiment, objects *N*, images *M*, and iterations *I* were set as 10, 10, and 300, respectively. We performed steps 1–5 with 10 trials for a statistical evaluation.

#### 4.1.2. Capturing and Memorizing Images (Step 1)

**Figure 7:**
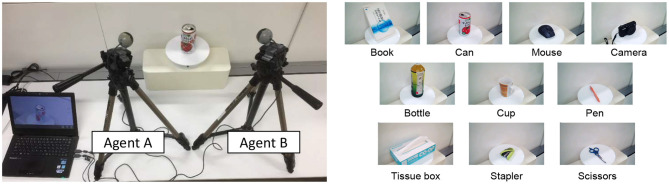
Experimental environment: two cameras were set as agent A and B with different angles. Each agent captured a target object placed at the center of two agents 10 times, and we prepared ten objects as targets.

Figure 7 shows the experimental environment. Two cameras on agents A and B captured object's images from different angles. Captured images were memorized on a computer. Resolution of a captured image was 640 pixels on width and 360 pixels on height. Target objects were a book, can, mouse, camera, bottle, cup, pen, tissue box, stapler, and scissors, as shown in the right side of Figure 7.

#### 4.1.3. Converting Memorized Images to Observations (Step 2)

**Figure 8:**
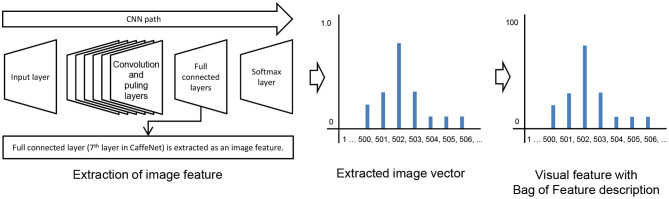
Conversion process from an image to a visual feature with Bag of Feature description.

An object's image captured by a camera is converted to a visual feature as an observation by CaffeNet (Jia et al., 2014), which is a framework for convolutional neural networks (CNN) (Krizhevsky et al., 2012) provided by Berkeley Vision and Learning Center. The parameters of CNN were trained by using the dataset from the ImageNet Large Scale Visual Recognition Challenge 2012^1^. In this study, CaffeNet is used as an image feature extractor. Figure 8 shows the conversion process from an image to a visual feature. In CNN, it is known that as the layer closer to the input layer approaches the output layer, it is gradually structured from low-order image features into semantic features specific to the dataset (Zeiler and Fergus, 2014).

Therefore, layers that are extremely low cannot benefit from the high discriminatory structure in CNN. In contrast, in a layer that is too high, it becomes too specialized for the training data set, and the generalization performance will be reduced. Therefore, the 6th or 7th layer, which are full connected layers one or two levels before the output layer, are generally used as image features in CaffeNet (Donahue et al., 2013; Matsukawa and Suzuki, 2016). In this study, the 7_*th*_ layer was empirically used as an image feature vector. The image feature vector is described by a 4,096-dimensional vector. This vector is multiplied by a constant to approximate the frequency of 4,096-dimensional Bag of Feature description as the visual feature of observation. Visual feature *o*_*i*_ ∈ {*o*_1_, *o*_2_, ⋯ , *o*_*I*_} was calculated by the following equation:

(16) $$\begin{array}{l} o_{i}=\frac{\exp\left(v_{i}\right)}{\sum_{j=0}^{I}\exp\left(v_{j}\right)}\times 10^{2}, \end{array}$$

where *v*_*i*_ ∈ {*v*_1_, *v*_2_, ⋯ , *v*_*I*_} is a value in the 7_*th*_ layer of CaffeNet. *I* is the number of output units at the 7_*th*_ layer and was set as 4096 in the experiment.

#### 4.1.4. Communication and Categorization (Steps 3 and 4)

To evaluate the effect of the proposed algorithm on the symbol emergence between agents and the categorization of each agent, we designed no communication and no rejection as the baseline communication algorithms. We conducted the experiment by employing the proposed model with three communication algorithms:

- No communication: *z* = 0 is used instead of Formula (10) in the inference. In this algorithm, two agents have no communication. Each agent does the categorization based on observations only, without word information from the other agent.
- No rejection: *z* = 1 is used instead of Formula (10) in the inference. In this algorithm, two agents accept all words from the other agent and update model parameters.
- Proposed algorithm: Formula (10) by the M-H algorithm is used in the inference. In this algorithm, two agents decide whether to accept or reject a word from the other agent based on self-organized categories.

The validity of the proposed model in the dynamics of symbol emergence and category formation and the functions required for semiotic communication are discussed by comparing the results between the proposed algorithm and two baseline algorithms, i.e., no rejection and no communication.

The hyperparameters of the proposed model were set as follows: α^*A*^ = 0.01, α^*B*^ = 0.001, β^*A*^ = 0.01, β^*B*^ = 0.001. The number of data points *D* was 100. The number of categories and words were set as follows: *L* = 15, *K* = 15, to cover ten target objects. As characters corresponding to the word index, we used the following 15 characters: a, b, c, d, e, f, g, h, i, j, k, l, m, n, and o.

#### 4.1.5. Evaluation Criteria (Step 5)

We evaluated the performance of the proposed model of the symbol emergence and concept formation using the following metrics: the kappa coefficient (Cohen, 1960) of words and adjusted rand index (ARI) (Hubert and Arabie, 1985) of categories between the agents.

The kappa coefficient was used as an evaluation criteria indicating the coincidence of words between agents A and B. Kappa coefficient κ was calculated by the following equation:

(17) $$\begin{array}{l} \kappa =\frac{C_{o}-C_{e}}{1-C_{e}}, \end{array}$$

where *C*_*o*_ is the coincidence rate of words between agents, and *C*_*e*_ is the coincidence rate of words between agents by random chance. The kappa coefficient is judged as follows: Excellent: (κ > 0.8), Good: (κ > 0.6), Moderate: (κ > 0.4), Poor: (κ > 0.0) (Landis and Koch, 1977).

The ARI was used as an evaluation criteria indicating the coincidence of categories between agents A and B. The ARI was calculated by the following equation:

(18) $$\begin{array}{l} \text{ARI}=\frac{\text{Index}-\text{Expected Index}}{\text{Max Index}-\text{Expected Index}}. \end{array}$$

Welch's *t*-test was used for statistical hypothesis testing between the proposed algorithm and two baseline algorithms, i.e., no communication and no rejection.

### 4.2. Experimental Results

Table 2 shows the experimental results: the kappa coefficient and ARI for the proposed algorithm and two baseline algorithms, i.e., no communication and no rejection.

**Table 2:** Kappa coefficient on words and ARI on categories between agents A and B: the result is described with mean, standard deviation (SD), *p*-value, and *t*-test for three algorithms: no communication, no rejection, and the proposed algorithm.

	**Kappa coefficient**				**ARI**			
**Algorithm**	**Mean**	**SD**	***P*-value**	***T*-test**	**Mean**	**SD**	***P*-value**	***T*-test**
No communication	0.01	0.04	8.9 × 10^−18^	**	0.89	0.07	2.2 × 10^−1^	n.s.
No rejection	0.57	0.06	1.0 × 10^−9^	**	0.88	0.03	3.0 × 10^−3^	**
Proposed algorithm	**0.88**	0.05	–	–	**0.92**	0.02	–	-

In the t-test, **p < 0.01, n.s. (not significant) p ≥ 0.05.

For the kappa coefficient on words between agents A and B, the proposed algorithm obtained a higher value than the baseline algorithms, and there were significant differences between the proposed algorithm and baseline algorithms in the *t*-test. The result implies that the agents used the same words for observations with a very high coincidence (of 0.8 or more) in the proposed algorithm.

The ARI for the proposed algorithm was higher than for the baseline algorithms, and there were significant differences between the proposed algorithm and no rejection. In case of no rejection, the word has a negative effect on categorization between the agents, comparing with the result of no communication. On the other hand, in the proposed algorithm that stochastically accepts the other agent's word based on self-knowledge, the word positively acts on the categorization between agents. This result suggests that a rejection strategy in the semiotic communication works as an important function in the language evolution. Naturally, our result suggests it is biologically feasible and mathematically feasible.

**Figure 9:**
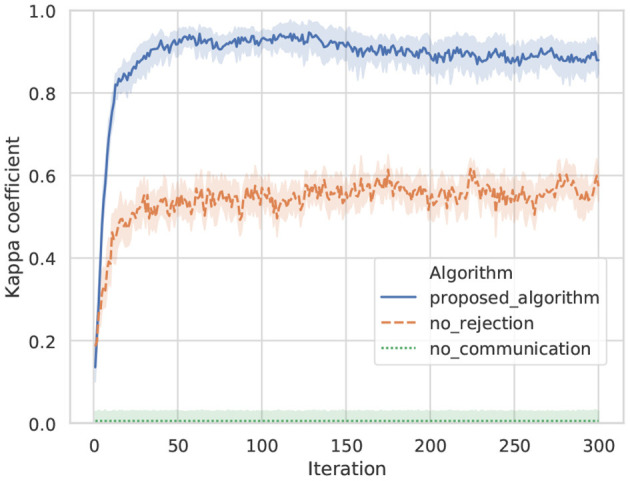
Transition on the kappa coefficient of words between agents: a line shows an average value, and top and bottom of each color show a maximum and minimum values in ten trials.

Figure 9 shows transitions in the kappa coefficients of words between agents A and B by the proposed algorithm, no rejection, and no communication in ten trials. In case of no communication, because all words of the opponent were rejected, the coincidence of words was at the level of random chance. In case of no rejection, although the coincidence of words is increasing in the initial iterations, it drifts and stagnates at ~0.55, which is a moderate value. In case of the proposed algorithm, the kappa coefficient is higher than for the baseline algorithms, and the sharing of words accompanying the increase in iterations was confirmed.

**Figure 10:**
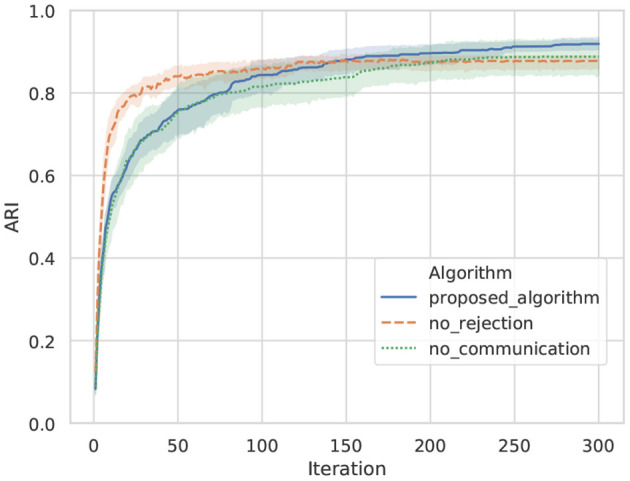
Transition on the ARI of categories between agents: a line shows an average value, and top and bottom of each color show a maximum and minimum values in ten trials.

Figure 10 shows transitions for the ARIs of categories between agents A and B by the proposed algorithm, no rejection, and no communication in ten trials. The ARI evaluates the similarity of categorization for object data among agents. A higher ARI indicates that similar categories are formed between agents. In this experiment, solely the viewpoint is different between agents. Therefore, it can be easily predicted that similar categories are formed between agents when categories are formed without semiotic communication. As a baseline, the ARI of no communication indicates that similar categories are formed between agents. However, in the result of no communication, words for sharing categories between agents are not formed. Words that share categories are formed in the proposed algorithm and no rejection. The ARI of the proposed algorithm is confirmed to exceed the ARI of no rejection in 150 iterations compared with the result of the proposed algorithm and no rejection. In the algorithm of no rejection, it is considered that learning progressed rapidly in the early part by utterances from other agents and their own observations, while utterances that did not match other agents for some observations caused confusion in category formation in the latter part. The proposed algorithm exhibited a higher ARI than no communication in the average, despite receiving utterances from other agents and sharing words, as well as no rejection. This indicates that the proposed algorithm effectively uses the utterances of others to form a common category among agents.

The result suggests the dynamics of symbol emergence and concept formation, where a symbol communication slowly affects the category formation in an agent and promotes sharing of the categories between the agents. It is a cognitively natural result: repetition of semiotic communication in the same environment gradually causes the sharing of categories between the agents.

**Figure 11:**
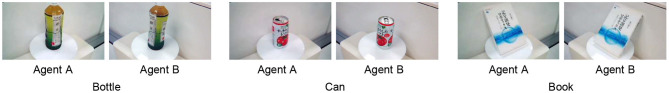
Examples of object's images: captured images of a bottle, can, and book from the viewpoints of agents A and B.

For qualitative evaluation, we showed words assigned to each of the three objects: bottle, can, and book. Figure 11 shows the examples of object's images observed from the viewpoints of agent A and B. Table 3 shows the words sampled by agents A and B for three example objects on three algorithms: the proposed algorithm and two baseline algorithms (no rejection and no communication). The sampled words are described as three best results out of ten sampled words for each object. (·) shows the rate of a word to ten sampled words. In case of no communication, a word representing an object was not shared between the agents. In case of no rejection, words representing an object, such as “i,” “b,” and “c” (for the bottle) were shared, but the probabilities of words are not high. In case of the proposed algorithm, it was confirmed that a word representing an object was shared between the agents with a high probability.

**Table 3:** Sampling results of words for three example objects by three communication algorithms, i.e., no communication, no rejection, and the proposed algorithm: the sampling results are described as 1_*st*_, 2_*nd*_, and 3_*rd*_ words in 10 sampled words for each object.

		**No communication**		**No rejection**		**Proposed algorithm**	
**Object**	**Word**	**Agent A**	**Agent B**	**Agent A**	**Agent B**	**Agent A**	**Agent B**
	1_*st*_	*h, l* (0.2)	*j, l* (0.2)	*b* (0.7)	*i* (0.5)	*c***(1.0)**	*c***(1.0)**
Bottle	2_*nd*_	–	–	*i* (0.2)	*b* (0.4)	–	–
	3_*rd*_	*b, d, f, i, m, o* (0.1)	*b, d, e, h, i, k* (0.1)	*c* (0.1)	*c* (0.1)	–	–
	1_*st*_	*h, i* (0.3)	*l* (0.3)	*j* (0.6)	*g* (0.5)	*f***(1.0)**	*f***(1.0)**
Can	2_*nd*_	–	*e* (0.2)	*g, k* (0.2)	*k* (0.3)	–	–
	3_*rd*_	*a, e, k, n* (0.1)	*b, g, h, j, o* (0.1)	–	j (0.2)	–	–
	1_*st*_	*m, o* (0.2)	*a* (0.3)	*n* (0.5)	*h* (0.4)	*b***(0.6)**	*b***(0.6)**
Book	2_*nd*_	–	*g, k* (0.2)	*c* (0.4)	*c, n* (0.3)	*k* (0.4)	*k* (0.4)
	3_*rd*_	*c, d, g, i, k, l* (0.1)	–	*h* (0.1)	–	–	–

*The rate of a word to 10 sampled words is described in (·)*.

**Figure 12:**
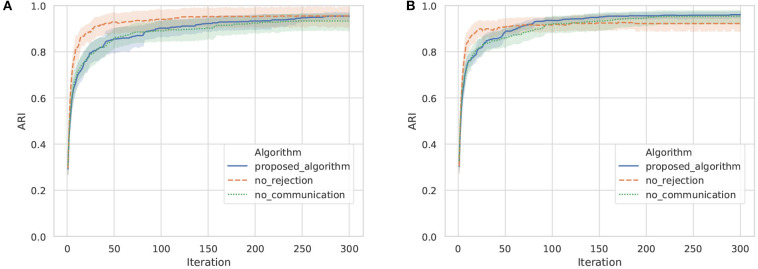
ARI between object labels and categories formed by the proposed algorithm in ten trials with agents A and B. The horizontal axis and vertical axis show the iteration and ARI, respectively. **(A)** Agent A, **(B)** Agent B.

To evaluate the accuracy of categorization of actual objects, the ARI between the object labels and categories formed by the proposed algorithm is shown in Figure 12. The ARIs in the three communication algorithms were above 0.9, which means that categories similar to actual objects have been formed in each agent. Focusing on the difference in the ARIs between agent A and B at the 300th iteration, the difference in no communication, no rejection, and the proposed algorithm was 0.023, 0.033, and 0.003, respectively. The ARI difference between agents in the proposed algorithm was small compared with the baseline algorithms. This is because the categorization results for objects are similar between agents by sharing categories through communication using words. This result demonstrates that the mechanism of category sharing through semiotic communication by the proposed algorithm works effectively, even in the categorization of actual objects.

**Figure 13:**
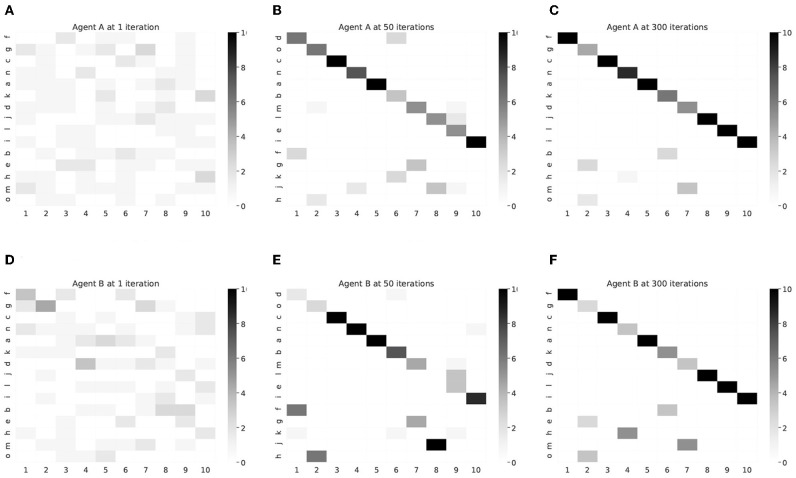
Confusion matrix between words and object's labels in each agent. The horizontal axis and vertical axis show the index of object's label and word, respectively. The order of the words was sorted according to the frequency of each object at agent A with 300 iterations. **(A)** Agent A, 1 iteration, **(B)** Agent A, 50 iterations, **(C)** Agent A, 300 iterations, **(D)** Agent B, 1 iteration, **(E)** Agent B, 50 iterations, **(F)** Agent B, 300 iterations.

The learning process of the correspondence relationship between words and objects for each agent is shown in Figure 13 as a confusion matrix. As the number of iterations increases, words corresponding to object labels were learned from random to one-on-one relationship. Each word was allocated to describe an object at the result of 300 iterations.

**Figure 14:**
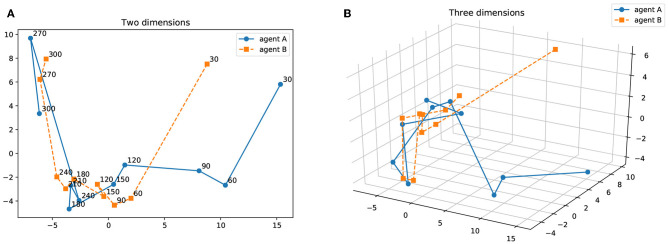
Results of PCA on the confusion matrix for agents A and B. The results are described from 30 to 300 iterations with a 30 iteration interval. **(A)** Two dimensions, **(B)** Three dimensions.

Figure 14 shows the result of principal component analysis (PCA) between the confusion matrices for agents A and B in Figure 13. The results are described from 30 to 300 iterations at 30 iterations intervals on two and three dimensions. As the number of iterations increases, the results of PCA on the confusion matrices of two agents are getting closer. This can be interpreted as a process in which the interpretation system of words and objects between the agents approaches by the iteration of the semiotic communication.

### 4.3. Discussion

We evaluated the validity of the proposed model and algorithm as a model of the dynamics on symbol emergence and category formation from the experiments using daily objects in the real-world environment. In the experiment, we compared the process of symbol emergence and category formation of objects between the agents by using three communication algorithms: the proposed algorithm, no rejection, and no communication. The experimental results demonstrated the following three events in the communication algorithms.

- In case of no communication, when the agent rejects all the other agent's utterances, the coincidence of categories was high but the coincidence of words was not shared between the agents. This result is understood as the following event: similar categories are formed when two agents have similar sensors that individually observe the same object.
- In case of no rejection, when the agent unconditionally accepts the other agent's utterances and updates the internal parameters, the coincidence of words drifts and stagnates, and the coincidence of categories decreases, compared with no communication. This result is understood as the following event: other agent's utterances that use different symbols interfere with categorization within the agent's individual as a noise.
- In the proposed algorithm, which probabilistically accepts the other agent's utterances based on the internal parameters, the coincidence of words was very high, and the coincidence of categories also had a high value compared with other algorithms. This result is also convincing as a mechanism of the symbol emergence and category formation based on the human semiotic communication.

Furthermore, it was suggested that the semiotic communication needs the function of rejecting other's utterances based on one's knowledge in the dynamics of symbol emergence and category formation between the agents. Evaluation of the validity of this suggestion in human semiotic communication will be a future work.

In addition, it is important whether the proposed model works in linguistic communication with practical robots and people. In the study of the hierarchical spatial concept (Hagiwara et al., 2018), the author implemented multimodal categorization based on feature extraction and mLDA model based on CNN, which is the basis of the proposed model. In this research, the robot [Toyota Human Support Robot (Yamamoto et al., 2019)] has achieved online learning through language instructions with a user in the practical world. The robot takes a few seconds for feature extraction and inference by GPU-equipped computer. Even in the proposed model of this study, there is no significant difference in the size of data used for feature extraction and inference, and it will scale to language learning using robots in the practical world. Additionally, in this study, CNN is used as a feature extractor for observations. Generally, the CNN needs training of a large-scale data set according to the target objects. However, feature vectors in an intermediate layer as used in this study are low-order image features that are less dependent on the target objects in the dataset and can be directly used for other objects and scenes as well.

## 5. Conclusions

This study focused on the symbol emergence in a multi-agent system and the category formation in individual agents through semiotic communication that is the generation and interpretation of symbols associated with categories formed from the agent's perception. We proposed a model and an inference algorithm representing the dynamics of symbol emergence and category formation through semiotic communication between the agents as an interpersonal multimodal categorizer. We showed the validity of the proposed model and inference algorithm on the dynamics of symbol emergence and concept formation in multi-agent system from the mathematical explanation and the experiment of object categorization and symbol emergence in a real environment. The experimental results on object categorization using three communication styles, i.e., no communication, no rejection, and the proposed algorithm based on the proposed model suggested that semiotic communication needs a function of rejecting other's utterances based on one's knowledge in the dynamics of symbol emergence and category formation between agents.

This study did not model an emergence of a grammar. However, the proposed model and algorithm succeeded in giving a mathematical explanation for the dynamics of symbol emergence in multi-agent system and category formation in individual agents through semiotic communication. This means our study showed a certain direction for treating multi-agent system logically in the symbol emergence and category formation.

As future work, we are extending the proposed model based on a mutual segmentation hypothesis of sound strings and situations based on co-creative communication (Okanoya and Merker, 2007).

The extension will be achieved through the following research process.

- The extension for a mutual segmentation model of sound strings and situations based on multimodal information will be achieved based on a mLDA with nested Pitman-Yor language model (Nakamura et al., 2014) and a spatial concept acquisition model that integrates self-localization and unsupervised word discovery from spoken sentences (Taniguchi A. et al., 2016).
- To reduce development and calculation costs associated with the large-scale model, “Serket: An Architecture for Connecting Stochastic Models to Realize a Large-Scale Cognitive Model” (Nakamura et al., 2018), will be used.
- Experiment with *N* agents will be performed on symbol emergence and concept formation by expanding the proposed model. We can design an experiment as a communication structure based on human conversation, because human conversation is usually performed by two people. In a related study, Oshikawa et al. (2018) proposed a Gaussian process hidden semi-Markov model, which enables robots to learn rules of interaction between persons by observing them in an unsupervised manner.
- Experimental results have shown the importance of a rejection strategy, but the evidence for the human brain to use such a strategy is not shown. We are planning to conduct psychological experiments.
- As an exploratory argument, mapping category *c* to observation *o* is theoretically possible for a neural network. A future study can develop a deep generative model, which integrates deep learning and generative model, by application of multimodal learning with deep generative models (Suzuki et al., 2016).

## Data Availability Statement

The datasets generated for this study are available on request to the corresponding author.

## Author Contributions

YH designed the study and wrote the initial draft of the manuscript. HK and AT contributed to analysis and interpretation of data, and assisted in the preparation of the manuscript. TT has critically reviewed the manuscript. All authors approved the final version of the manuscript, and agree to be accountable for all aspects of the work in ensuring that questions related to the accuracy or integrity of any part of the work are appropriately investigated and resolved.

### Conflict of Interest

The authors declare that the research was conducted in the absence of any commercial or financial relationships that could be construed as a potential conflict of interest.

## Footnotes

^1^ILSVRC2012: http://www.image-net.org/challenges/LSVRC/2012/
