# Supplementary material for: Symbol Emergence as an Interpersonal Multimodal Categorization
Source: Front Robot AI. 2019 Dec 10;6:134. doi: 10.3389/frobt.2019.00134 (PMC7805687; doi:10.3389/frobt.2019.00134)
Supplement: Supplementary file 1 [file Data_Sheet_1.PDF]

Supplementary Material:
Symbol Emergence as an Interpersonal Multimodal Categorization

1 SUPPLEMENTARY FORMULAS

This subsection describes the details of calculation process in the proposed inference algorithm.

On the sampling and judgment of words W with an agent as the speaker and the other agent as the listener, the model parameters θ_k, ϕ_l, c_d and w_d are inferred with the following process.

- Step 1: sampling word w_d^{Sp} for a data d at an iteration t based on the parameters of the speaker by the following formulas:

$$\begin{aligned} \phi_l^{Sp} &\sim P(\phi_l^{Sp} \mid \mathbf{C}^{Sp}, \mathbf{O}^{Sp}, \beta^{Sp}) \\ &\propto \mathrm{Multi}(\mathbf{O}_l^{Sp} \mid \phi_l^{Sp})\mathrm{Dir}(\phi_l^{Sp} \mid \beta^{Sp}) \\ &\propto \mathrm{Dir}(\phi_l^{Sp} \mid \mathbf{O}_l^{Sp}, \beta^{Sp}), \end{aligned} \tag{S1}$$

  where $\mathbf{C}^{Sp}$ denotes a set of all the indices of categories in the speaker, $\mathbf{O}^{Sp}$ denotes a set of all the observed features in the speaker, and $\mathbf{O}_l^{Sp}$ denotes a set of all the observed features for the category $c_d^{Sp} = l$ in $d \in \{1, 2, \ldots, D\}$.

$$\begin{aligned} \theta_k^{Sp} &\sim P(\theta_k^{Sp} \mid \mathbf{W}, \mathbf{C}^{Sp}, \alpha^{Sp}) \\ &\propto \mathrm{Multi}(\mathbf{C}_k^{Sp} \mid \theta_k^{Sp})\mathrm{Dir}(\theta_k^{Sp} \mid \alpha^{Sp}) \\ &\propto \mathrm{Dir}(\theta_k^{Sp} \mid \mathbf{C}_k^{Sp}, \alpha^{Sp}), \end{aligned} \tag{S2}$$

$$\begin{aligned} c_d^{Sp} &\sim P(c_d^{Sp} \mid w_d, \theta_k^{Sp}, o_d^{Sp}, \phi_l^{Sp}) \\ &\propto \mathrm{Multi}(c_d^{Sp} \mid \theta_{w_d}^{Sp})\mathrm{Multi}(o_d^{Sp} \mid \phi_{c_d^{Sp}}^{Sp}), \end{aligned} \tag{S3}$$

  where $\mathbf{W}$ denotes a set of all the words, and $\mathbf{C}_k^{Sp}$ denotes a set of all the indices of categories for the word $w_d^{Sp} = k$ in $d \in \{1, 2, \ldots, D\}$.

$$w_d^{Sp} \sim P(w_d^{Sp} \mid c_d^{Sp}, \theta_k^{Sp}), \tag{S4}$$

  where word w_d^{Sp} for a data d is sampled from the proposed distribution $P(w_d^{Sp} \mid c_d^{Sp}, \theta_k^{Sp})$, which can be calculated as the conditional probability of a category c_d^{Sp} and parameter θ_k^{Sp} in the agent A.
- Step 2: calculate the acceptance rate z based on parameters on agent B by the following formulas:

$$
\begin{aligned}
z &\sim \min\left(1, \frac{P(w_d^{Sp} \mid c_d^{Sp}, c_d^{Li}, \theta_k^{Sp}, \theta_k^{Li})P(w_d^{Li}) \mid c_d^{Sp}, \theta_k^{Sp})}{P(w_d^{Li}) \mid c_d^{Sp}, c_d^{Li}, \theta_k^{Sp}, \theta_k^{Li})P(w_d^{Sp} \mid c_d^{Sp}, \theta_k^{Sp})}\right) \\
&\propto \min\left(1, \frac{P(c_d^{Sp} \mid \theta_k^{Sp}, w_d^{Sp})P(c_d^{Li} \mid \theta_k^{Li}, w_d^{Sp})P(w_d^{Sp})P(c_d^{Sp} \mid \theta_k^{Sp}, w_d^{Li})P(w_d^{Li})}{P(c_d^{Sp} \mid \theta_k^{Sp}, w_d^{Li})P(c_d^{Li} \mid \theta_k^{Li}, w_d^{Li})P(w_d^{Li})P(c_d^{Sp} \mid \theta_k^{Sp}, w_d^{Sp})P(w_d^{Sp})}\right) \\
&\propto \min\left(1, \frac{P(c_d^{Li} \mid \theta_k^{Li}, w_d^{Sp})}{P(c_d^{Li} \mid \theta_k^{Li}, w_d^{Li})}\right).
\end{aligned}
\tag{S5}
$$

- Step 3: decide to accept or reject w_d^{Sp} based on the acceptance rate z by the following formulas:

$$
u \sim \mathrm{Unif}(0, 1), \tag{S6}
$$

where the continuous uniform distribution is denoted as $\mathrm{Unif}(\cdot)$.

$$
w_d^{[i]} = \begin{cases} w_d^{Sp[i]} & (u \leq z) \\ w_d^{Li[i-1]} & (otherwise). \end{cases} \tag{S7}
$$

- Step 4: update parameters on the listener if w_d^{Sp} is accepted by the following formulas:

$$
\begin{aligned}
\phi_l^{Li} &\sim P(\phi_l^{Li} \mid \mathbf{C}^{Li}, \mathbf{O}^{Li}, \beta^{Li}) \\
&\propto \mathrm{Multi}(\mathbf{O}_l^{Li} \mid \phi_l^{Li})\mathrm{Dir}(\phi_l^{Li} \mid \beta^{Li}) \\
&\propto \mathrm{Dir}(\phi_l^{Li} \mid \mathbf{O}_l^{Li}, \beta^{Li}),
\end{aligned}
\tag{S8}
$$

$$
\begin{aligned}
\theta_k^{Li} &\sim P(\theta_k^{Li} \mid \mathbf{W}, \mathbf{C}^{Li}, \alpha^{Li}) \\
&\propto \mathrm{Multi}(\mathbf{C}_k^{Li} \mid \theta_k^{Li})\mathrm{Dir}(\theta_k^{Li} \mid \alpha^{Li}) \\
&\propto \mathrm{Dir}(\theta_k^{Li} \mid \mathbf{C}_k^{Li}, \alpha^{Li}),
\end{aligned}
\tag{S9}
$$

$$
\begin{aligned}
c_d^{Li} &\sim P(c_d^{Li} \mid w_d, \theta_k^{Li}, o_d^{Li}, \phi_l^{Li}) \\
&\propto \mathrm{Multi}(c_d^{Li} \mid \theta_{w_d}^{Li})\mathrm{Multi}(o_d^{Li} \mid \phi_{c_d^{Li}}^{Li}),
\end{aligned}
\tag{S10}
$$

- Repeating steps 1 to 4 with I iterations while exchanging the speaker and the listener.
